# Acupuncture improves immunity and fatigue after chemotherapy in breast cancer patients by inhibiting the Leptin/AMPK signaling pathway

**DOI:** 10.1007/s00520-023-07967-1

**Published:** 2023-08-05

**Authors:** Jinxia Li, Ruiyang Fu, Xiaoqing Guo, Zhongqiang Pan, Jingjun Xie

**Affiliations:** 1 Department of Acupuncture, Huzhou Hospital of Traditional Chinese Medicine Affiliated to Zhejiang Chinese Medical University, Huzhou, 313000 Zhejiang People’s Republic of China; 2 Department of Rehabilitation Medicine, The First People’s Hospital of Huzhou, No. 158, Guangchang Hou Road, Huzhou, 313000 Zhejiang People’s Republic of China

**Keywords:** Breast cancer, Post-chemotherapy fatigue, Acupuncture treatment, Leptin, AMPK, Mitochondrial functional impairment

## Abstract

**Objective:**

Acupuncture has become a popular complementary treatment in oncology. This study is based on RNA-Seq transcriptome sequencing technology to investigate the molecular mechanisms underlying the effect of acupuncture-mediated regulation of the Leptin/AMPK signaling pathway on mitochondrial dysfunction-induced fatigue in breast cancer patients after chemotherapy.

**Methods:**

Peripheral blood samples from 10 patients with post-operative chemotherapy for breast cancer were selected for transcriptome sequencing to screen the key molecular pathways involved in fatigue after chemotherapy in breast cancer patients. Besides, peripheral blood samples were collected from 138 post-operative chemotherapy patients with breast cancer to study the composite fatigue and quality of life scores. Flow cytometry was used to detect T lymphocyte subsets in peripheral blood-specific immune cells. In addition, a blood cell analyzer was used to measure peripheral blood leukocyte counts, and MSP-PCR was used to detect mitochondrial DNA mutations in peripheral blood leukocytes.

**Results:**

Transcriptome bioinformatics analysis screened 147 up-regulated mRNAs and 160 down-regulated mRNAs. Leptin protein was confirmed as the key factor. Leptin was significantly higher in the peripheral blood of breast cancer patients who developed fatigue after chemotherapy. Acupuncture treatment effectively improved post-chemotherapy fatigue and immune status in breast cancer patients, suppressed the expression of Leptin/AMPK signaling pathway-related factor and leukocyte counts, and significantly reduced the rate of mitochondrial DNA mutations in peripheral blood leukocytes.

**Conclusion:**

The Leptin/AMPK signaling pathway may be the key molecular pathway affecting the occurrence of fatigue after chemotherapy in breast cancer patients. Leptin may improve post-chemotherapy fatigue in breast cancer patients by activating AMPK phosphorylation and alleviating mitochondrial functional impairment.

**Supplementary Information:**

The online version contains supplementary material available at 10.1007/s00520-023-07967-1.

## Introduction

Breast cancer is the second-leading cause of cancer-related deaths globally and the most prevalent malignancy in women [1, 2]. For the first time, breast cancer in women will surpass lung cancer as the most commonly diagnosed cancer, accounting for 11.7% of new cancer cases [3]. The World Health Organization (WHO) reports that breast cancer accounts for 11.7% of all cancer cases globally [4]. The incidence rate of breast cancer varies globally, with developed countries typically having a high incidence rate [5]. The USA, Canada, Australia, New Zealand, and Western European countries have a high incidence rate of breast cancer, while Asia, Africa, and Latin America have a low incidence rate [6].

The main treatments for breast cancer are currently surgery, radiotherapy, targeted therapy, and immunotherapy [7]. Surgery is the preferred treatment for breast cancer, and chemotherapy is the most commonly used cancer treatment other than surgery and is the preferred treatment for breast cancer patients [8]. Researchers proposed cancer-related fatigue (CRF) in the late 1970s [9]. Subsequent investigations have established it as a multifaceted and individualized negative subjective experience. CRF is known to be influenced by various factors, including physical, mental, psychological, and socio-cultural backgrounds [10]. Previous studies have demonstrated that fatigue affects over 83% of cancer patients, with its severity often exacerbated by radiotherapy [11]. Considering the essential role of chemotherapy in cancer treatment, fatigue has emerged as a significant disruption to patients’ daily lives.

CRF has been reported to occur in up to 99% of patients during chemotherapy [12]. The NCCN (National Comprehensive Cancer Network) defines cancer-related fatigue as a distressing, subjective feeling of fatigue or exhaustion that interferes with normal life [13]. It has been suggested that CRF is the most distressing and severe symptom felt by patients compared to other adverse effects of cancer treatment (e.g., nausea, pain, and peripheral nerve symptoms) [14]. In addition, studies have identified bodily, psychological, therapeutic, and social factors as the main factors influencing CRF, which are generally consistent with the NCCN’s top five associated factors [15].

Leptin is a pleiotropic peptide hormone [16] produced by tissues such as fat and cartilage. Early studies found that it mainly affects energy metabolism in the central system [17]. Still, now it has been found that Leptin also has a dual role as a hormone and cytokine, participating in the regulation of several processes such as the inflammatory response, immune homeostasis, cartilage and bone metabolism, bone formation, angiogenesis, wound healing, and intestinal nutrient absorption [18]. Leptin was closely associated with post-chemotherapy fatigue in breast cancer patients. Leptin expression was elevated in breast cancer patients who developed post-chemotherapy fatigue, suggesting that Leptin is a potential biomarker of post-chemotherapy fatigue in adenocarcinoma and that the mechanism may be that high Leptin expression activates TNF-α, thereby promoting post-chemotherapy fatigue in adenocarcinoma [19].

AMP-activated protein kinase (AMPK) is an evolutionarily conserved serine/threonine kinase that induces metabolic changes to maintain the balance of cell energy production and consumption [20]. AMPK is closely associated with developing diseases such as cancer, pulmonary hypertension, type 2 diabetes, cerebral hemorrhage, and myocarditis [21]. It has also been found that several targets of AMPK are associated with mitochondrial homeostasis [22]. In addition, it was found that targeted inhibition of the AMPK/PI3K/Akt signaling pathway could inhibit myocyte apoptosis, increase myocyte mitochondrial membrane potential, and reduce mitochondrial functional impairment, thus improving post-chemotherapy fatigue [23].

Acupuncture is increasingly recognized as a complementary therapeutic strategy for CRF. The National Comprehensive Cancer Network (NCCN) guidelines in the USA recommend acupuncture for CRF patients, especially cancer survivors who have completed anticancer treatments. A randomized controlled trial (RCT) has also been conducted to evaluate acupuncture’s efficacy and safety in treating chronic renal failure [24]. In this study, we investigated the key molecular pathways through which Leptin regulates the AMPK signaling pathway to influence the onset of post-chemotherapy fatigue in breast cancer patients. We tentatively concluded that Leptin may induce AMPK phosphorylation activation. When the Leptin/AMPK signaling pathway was inhibited by acupuncture treatment, it could reduce mitochondrial functional impairment and thus improve post-chemotherapy fatigue in breast cancer patients. It also makes factors such as Leptin and AMPK targets for improving post-chemotherapy fatigue in adenocarcinoma patients, thus providing a new theoretical basis for improving post-chemotherapy fatigue in adenocarcinoma patients with acupuncture treatment.

## Materials and methods

### Clinical sample collection

One hundred and thirty-eight patients with post-operative chemotherapy for breast cancer in our hospital were collected and randomly divided into 68 cases in the acupuncture treatment group and 70 cases in the sham acupuncture treatment group (given sham acupuncture treatment). In addition to the conventional symptomatic treatment (including blood transfusion, anti-pain, anti-emetic, anti-cough, and phlegm), the patients in the acupuncture treatment group were given multiple acupuncture points after chemotherapy for 2 weeks. The specific methods of the acupuncture treatment group were as follows: after routine disinfection of the local skin at the acupoints, acupuncture needles were used to directly pierce Guan Yuan, Sanyinjiao, Sansili, Qi Hai, Blood Sea, and Neiguan points. After obtaining Qi, the needles were retained for 20 min, and the treatment was performed once every 10 min, once daily for 2 weeks [25]. Patients were given conventional symptomatic support and sham acupuncture treatments in the sham acupuncture group. Sham acupuncture procedure: During the sham acupuncture treatment, the therapeutic needle is inserted approximately 1 inch away from the actual acupuncture point, while the remaining procedures are consistent with the verum acupuncture treatment [26]. Patients in both groups were given conventional education, psychological support, sleep counseling, and exercise instruction.

Inclusion criteria: (i) meet the above Chinese and Western medical diagnostic criteria; (ii) age 18–75 years; (iii) expected survival ≥ 3 months; (iv) QLQ-C30 score ≥ 60 and moderate fatigue or above by the PIPER Fatigue Revision Scale; (v) receive chemotherapy during this treatment; (vi) clear consciousness, no intellectual impairment, able to understand the content of the scale; (vii) voluntary participation and signed informed consent form. Exclusion criteria: (i) those who do not meet the inclusion criteria; (ii) critically ill with survival < 3 months; (iii) with serious primary diseases such as liver, kidney, cardiovascular, or hematopoietic system; (iv) severe cognitive impairment or psychiatric disorders; (v) ulceration or infection at the needle site [27].

### RNA extraction and sequencing

Ten peripheral blood samples from patients undergoing chemotherapy after breast cancer surgery were selected for transcriptome sequencing, including five patients who developed fatigue after chemotherapy (observation group) and five patients who did not develop fatigue after chemotherapy (control group). Total RNA was isolated using Trizol reagent (Invitrogen, USA), and RNA sample concentrations were determined by OD260/280 using a Nanodrop ND-1000 spectrophotometer (Thermo Fisher Scientific, USA) instrument. RNA concentrations were measured using a Qubit RNA analysis kit. Total RNA samples met the following requirements for subsequent experiments: RNA integrity index (RIN) ≥ 7.0, 28S:18S ratio ≥ 1.5.

Sequencing libraries were generated and sequenced by CapitalBio Technology (Beijing, China). A total of 5 μg RNA was used per sample. Briefly, ribosomal RNA (rRNA) was removed from total RNA using the Ribo-Zero™ Magnetic Kit (Epicentre Technologies, Madison, Wisconsin, USA). Sequencing libraries were constructed using Illumina’s NEB Next Ultra RNA Library Preparation Kit (NEB, USA). The RNA was then fragmented into fragments of approximately 300 base pairs (bp) in length in NEB Next First Strand Synthesis Reaction Buffer (5 ×). First-strand cDNA was synthesized using reverse transcriptase primers and random primers, and second-strand cDNA was synthesized in second-strand synthesis reaction buffer in dUTP Mix (10 ×). End repair of cDNA fragments, including adding poly(A) tails and ligating sequencing junctions. After ligating the Illumina sequencing junction, the second strand of cDNA was digested using USER Enzyme (NEB, USA) to construct a strand-specific library. The library DNA was amplified, and the library was purified and enriched by PCR. Libraries were then identified by Agilent 2100 and quantified using the KAPA Library Quantification Kit (KAPA Biosystems, South Africa). Finally, paired-end sequencing was performed on an Illumina NextSeqCN500 sequencer.

### Sequencing data quality control and reference genome alignment

Using FastQC software v0.11.8 (http://www.bioinformatics.babraham.ac.uk) to check the quality of paired-end reads of raw sequencing data. Using Cutadapt software 1.18 (http://www.bioinformatics.babraham.ac.uk) to pre-process the raw data: remove Illumina sequencing junctions and poly(A) tail sequences. Reads with more than 5% N content were removed by Perl scripts. Reads were removed using FASTX Toolkit software 0.0.13 (http://hannonlab.cshl.edu/fastx_toolkit/) to extract 70% of reads with base masses above 20. Reads with N content above 20 were extracted using the BBMap software (https://sourceforge.net/projects/bbmap/). The double-ended sequences were repaired. Finally, the filtered fragments of high-quality reads were compared to the reference genome by hisat2 software (0.7.12).

### Bioinformatics analysis to screen for differentially expressed genes

The mRNA-based read counts were analyzed using the “edgeR” package in the R language for differential expression analysis of mRNA. The criteria for screening differentially expressed genes were set as |log2 FC|> 1 and *P*-value < 0.05. KEGG pathway enrichment analysis was performed using the “ClusterProfiler” R software package (https://cytoscape.org/) for differentially expressed genes, with statistical significance defined at *P* < 0.05. The STRING database (https://string-db.org/) was utilized for protein interaction analysis between genes, while Cytoscape software (https://cytoscape.org/) was employed for visualizing the protein–protein interaction (PPI) network. Gene interaction analysis was conducted using the GeneMANIA database (http://genemania.org/), and gene correlation analysis was performed using the GEPIA database (http://gepia.cancer-pku.cn/).

### Symptom assessments between the two groups before and after acupuncture treatment

The blinded methodology was implemented as follows: Both patients and assessors were unaware of group assignments. Under this condition, assessors interviewed the patients to evaluate the relevant outcomes [28]. The fatigue assessment tools currently applied in TCM research include the Brief Fatigue Inventory (BFI), the Multidimensional Fatigue Inventory (MFI-20), the PIPER Fatigue Scale-Revised (PFSR), and the Cancer Fatigue Scale (CFS). The PIPER Fatigue Assessment Scale-Revised (PFSR), which was revised from the original scale by Piper et al. in a study of women with breast cancer in 1998, with a Cronbach’s alpha coefficient of 0.97. The PFSR was used to assess cancer-caused fatigue, with a total of 22 entries and a score out of 10, where 0 is no fatigue; scores of 1 to 3 indicate mild fatigue; 4 to 6 indicate moderate fatigue; and 7 to 10 represent severe fatigue. The scale consists of 4 items: behavioral, emotional, sensory, and cognitive, with higher scores indicating more severe fatigue [29].

The patient’s quality of life is evaluated using the QLQ-C30 Quality of Life Score, a 30-item scale divided into 15 domains. It includes 5 functional domains (physical, role, cognitive, emotional, and social functioning), 3 symptom domains (fatigue, pain, nausea, and vomiting), 1 general health status domain, and 6 individual items (shortness of breath, insomnia, loss of appetite, constipation, diarrhea, financial difficulties). The raw score (RS) is calculated by adding the scores of the items in each domain and dividing them by the number of items included in that domain. The raw score is then linearly transformed by polarization into a standardized score (SS) of 0 to 100 to facilitate domain comparison. For the functional domain and general health, higher scores indicate better functioning or health; for the symptom domain, higher scores on individual items indicate more severe symptoms or problems. We scored each of the four dimensions reflecting the quality of life separately, with higher scores for each function and overall quality of survival in both groups suggesting the better quality of life and higher scores for fatigue suggesting the worse quality of life [30].

### Detection of T lymphocyte subsets in peripheral blood-specific immune cells by flow cytometry

NK cell counts and T lymphocyte subsets were analyzed in peripheral blood before and after treatment using a flow cytometer (FAC-SC-ALIBUR, Becton Dickinson, USA).

### Methods

Peripheral blood should be removed by adding erythrocyte lysate, collecting cells by centrifugation (2990 r/min for 5 min), and adding anti-CD2-PE (anti-CD phaeohemoglobin, eBioscience, USA) and anti-CD&-FITC or anti-CDa-FITC (anti-CD-isothiocyanate). After mixing, the cells were placed at 4.0 °C for 30 min protected from light and washed twice with phosphate buffer (PBS) (2990 r/min, 5 min) and detected and analyzed by flow cytometry. The obtained data were acquired by FACSC-ALI-BUR flow cytometer and Cellquest software. First, the lymphocyte region R1 was plotted in a front scatter (FSC) versus side scatter (SSC) two-dimensional scatter plot, and then the lymphocyte FITC and PE fluorescence intensities were detected. The FITC is fluorescence 1 (FL1), and the PE is fluorescence 2 (FL2). Ten thousand cells per sample tube were assayed, and the data obtained were analyzed using Cell Quest software [31, 32].

### Detection of IgA, IgG, and IgM levels in peripheral blood by immunoturbidimetric assay

The serum was stored in a refrigerator at − 80 °C and kept to determine serum IgG, IgM, and IgA levels. Serum IgG, IgM, and IgA expression levels were determined by an immunoturbidimetric assay using IMMAGE800 specific protein instrument [33–35].

### ELISA for the expression of Leptin-AMPK signaling pathway-related factors in peripheral blood samples

Peripheral blood samples were collected from patients. Leptin-AMPK signaling pathway-related factors were quantified in peripheral blood samples using the Human Leptin ELISA Kit (PL700, Beyotime Biotechnology, Shanghai, China) assay kit. All indicators were analyzed according to the manufacturer’s specifications.

### RT-qPCR

Total RNA was extracted from peripheral blood samples using TRIZOL reagent (15596–018, Solarbio, USA, https://www.solarbio.com/) according to the manufacturer’s instructions. To measure mRNA expression, total RNA was reverse transcribed into cDNA using the PrimeScript™ RT-PCR kit (TaKaRa, USA). Total RNA was reverse transcribed into cDNA using the SYBR Premix Ex Taq™ (TaKaRa) for real-time quantification on a LightCycler 480 system (Roche Diagnostics, Pleasanton, CA, USA). RT-qPCR was performed to measure the gene expression of Leptin and AMPK, with GAPDH serving as the internal reference gene for mRNA expression level normalization. The primers used for amplification were designed and synthesized by Shanghai General Biotechnology Co. The primer sequences are shown in Table S1. The relative transcript levels of the target genes were calculated using the relative quantification method (2^−△△^ CT method). Three replicate wells were set up for each sample, and each experiment was repeated three times.

### Western blot

Western blot analysis was employed to examine the protein levels of Leptin and AMPK. Total protein was extracted using enhanced RIPA lysate containing 1% protease inhibitor (P0013B, Beyoncé Biotechnology Co., Ltd., Shanghai, China, http://beyotime.bioon.com.cn/reagent_32771.html) according to strict instructions and protein was measured using the Beyoncé BCA protein quantification kit concentration. SDS-PAGE separated the proteins, and the separated proteins were electrotransferred to PVDF membranes, and 5% BSA was closed at room temperature for 1 h. Primary antibodies for Leptin (ab16227, 1:1000 dilution, rabbit antibody, Abcam, UK) and AMPK (ab32047, 1:2000 dilution, rabbit antibody, Abcam, UK) were applied to the sample. Following this, the membranes were washed three times with PBST, each wash lasting 5 min. After washing, the anti-rabbit-HRP secondary antibody (Cat # 7074, 1:5000 dilution; CST, USA) was added and allowed to interact for 1 h at room temperature. This was followed by another three rounds of washing with PBST, each round lasting 5 min. Throughout the process, GAPDH was used as the control. The PBST was discarded, an appropriate amount of ECL working solution (CPSOC, Sigma) was added, and the transfer film was incubated at room temperature for 1 min. The excess ECL reagent was removed, the film was sealed with cling film, and an X-ray film (Z380164, Sigma) was placed in the dark box for 5–10 min after exposure for development and fixation. The groups of bands in the Western blot images were quantified in greyscale using Image J analysis software, with GAPDH as an internal reference.

### Blood cell analyzer for peripheral blood leukocyte count

#### Sample processing

Venous blood was drawn from all enrolled observers on an empty stomach in the morning. Peripheral blood leukocyte (WBC) count was completed using a SysmexXT-4000i blood cell analyzer [36].

### MSP-PCR detection of mitochondrial DNA mutations in peripheral blood leukocytes

To extract mtDNA from peripheral blood leukocytes, 2–3 ml of heparin-anticoagulated venous blood was extracted from each leukocyte and purified the mtDNA. GATCCTTGCATGTGTAATCT-3′, the primers were synthesized by the Shanghai Bioengineering Research Centre of the Chinese Academy of Sciences and purified by PAGE. The high-fidelity PCR kit and PCR product purification kit were purchased from Roche. The 1528 bp PCR product nucleic acid fragment was used as the sequencing template. The sequences were sequenced automatically on an ABI prism 3700 sequencers using the dideoxytetra-color fluorescent dye labeling method. For statistical processing, sequencing data were analyzed using DNAStar software. Sequences were proofread using the GenBank H. sapiens mitochondrial genome version with base variation rate = the total number of variant bases/the total number of bases measured × 100% [37–39].

### Statistical methods

All data were analyzed using SPSS 24.0 statistical software (SPSS, Inc., USA). Normality and homogeneity of variance tests were conducted for all data. Normally distributed continuous variables are presented as mean ± standard deviation. Independent samples *t*-test was used for comparisons between the two groups. A significance level of *P* < 0.05 was considered statistically significant for all analyses.

## Results

### Leptin/AMPK signaling pathway identified using transcriptome sequencing

**Fig. 1:**
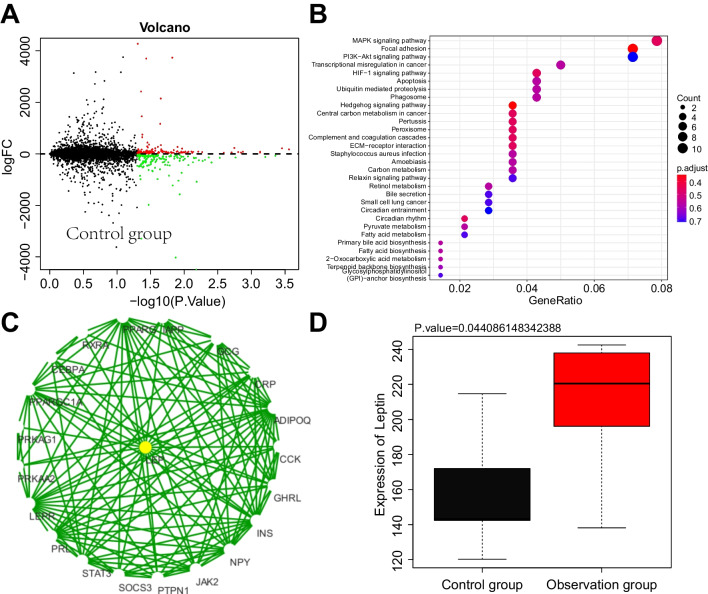
Key molecular pathways for screening the occurrence of fatigue in breast cancer patients after chemotherapy using transcriptome sequencing. Note: **A** volcano plot of differentially expressed genes (red indicates up-regulation, green indicates down-regulation, and black indicates no significant difference); **B** KEGG functional analysis of DEmRNAs (where gene color scales from blue to red represent log2FC values from negative to positive, and the size of the dots represents the number of selected genes); **C** PPI network diagram of candidate target genes; **D** box plot of Leptin expression in chip data (where black represents the control group, and red represents the observation group of breast cancer patients with fatigue after chemotherapy)

The identified mRNA counts were analyzed differently using the R language “edgeR” package, resulting in 147 up-regulated mRNAs and 160 down-regulated mRNAs (Fig. 1A). KEGG enrichment analysis showed that they were mainly involved in the AMPK/p38 MAPK signaling pathway, focal adhesion, PI3K-Akt signaling pathway and HIF-1 signaling pathway, and other biological processes (Fig. 1B). Further PPI analysis of differentially expressed genes revealed that Leptin was at the core of the PPI network (Fig. 1C). In addition, Leptin was significantly lower in the peripheral blood of the observation group (breast cancer patients who developed fatigue after chemotherapy) compared to the control group (breast cancer patients who did not develop fatigue after chemotherapy) (Fig. 1D).

### Leptin/AMPK signaling pathway may affect fatigue after chemotherapy in breast cancer patients

**Fig. 2:**
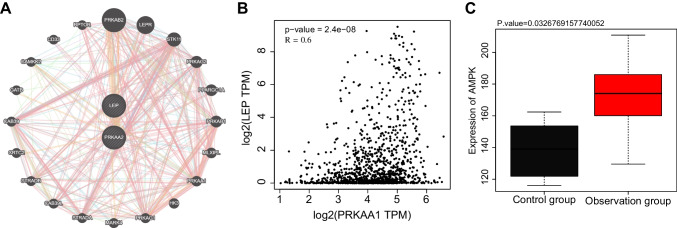
Analysis of the correlation between Leptin/AMPK and breast cancer patients in the TCGA database. Note: **A** GeneMANIA database predicts the Leptin/AMPK signaling pathway; **B** GEPIA analyzes the correlation between Leptin/AMPK (where the horizontal axis represents log2(PRKAA1 TPM), the vertical axis represents log2(LEP TPM), *P*-value < 0.05); **C** box plot of AMPK expression results in sequencing data (black represents the control group, breast cancer patients after chemotherapy without fatigue; red represents the observation group, breast cancer patients after chemotherapy with fatigue)

To investigate the molecular mechanisms that regulate fatigue after chemotherapy in breast cancer patients, we predicted through the GeneMANIA database that Leptin activates its downstream target gene AMPK (Fig. 2A). The correlation analysis with GEPIA showed that Leptin and AMPK were closely related (P< 0.05) (Fig. 2B). Meanwhile, the sequencing results showed that compared with the control group, the expression level of AMPK in the peripheral blood of patients in the observation group was also significantly increased (Fig. 2C). Combined with the results of differential gene KEGG enrichment analysis (Fig. 1B), AMPK is mainly involved in AMPK/p38 MAPK signaling pathway and other biological processes.

### Acupuncture improves post-chemotherapy fatigue and immune status in breast cancer patients

To investigate the effects of acupuncture treatment on fatigue symptoms and immune status of breast cancer patients before and after chemotherapy, we counted the PFSR fatigue scores and QLQ-C30 quality of life scores before and after acupuncture treatment in two groups of patients. As shown in Table S2, there was no statistically significant difference between the scores of each dimension and the total scores of the two groups before treatment (*P* > 0.05). However, when compared within the group before and after treatment, the differences in the scores of each dimension and total scores between the two groups were statistically significant (*P* < 0.05); when compared between the groups after treatment, the acupuncture treatment group was significantly lower than the sham acupuncture treatment group (*P* < 0.05). As shown in Table S3, the differences were not statistically significant (*P* > 0.05) when comparing the QLQ-C30 scores of each dimension between the two groups before treatment; when comparing between groups after treatment, the QLQ-C30 scores of each dimension in the acupuncture treatment group were significantly higher than those in the sham acupuncture treatment group (*P* < 0.05).

### Acupuncture increases Leptin/AMPK signaling pathway-related factors in breast cancer patients after chemotherapy

**Fig. 3:**
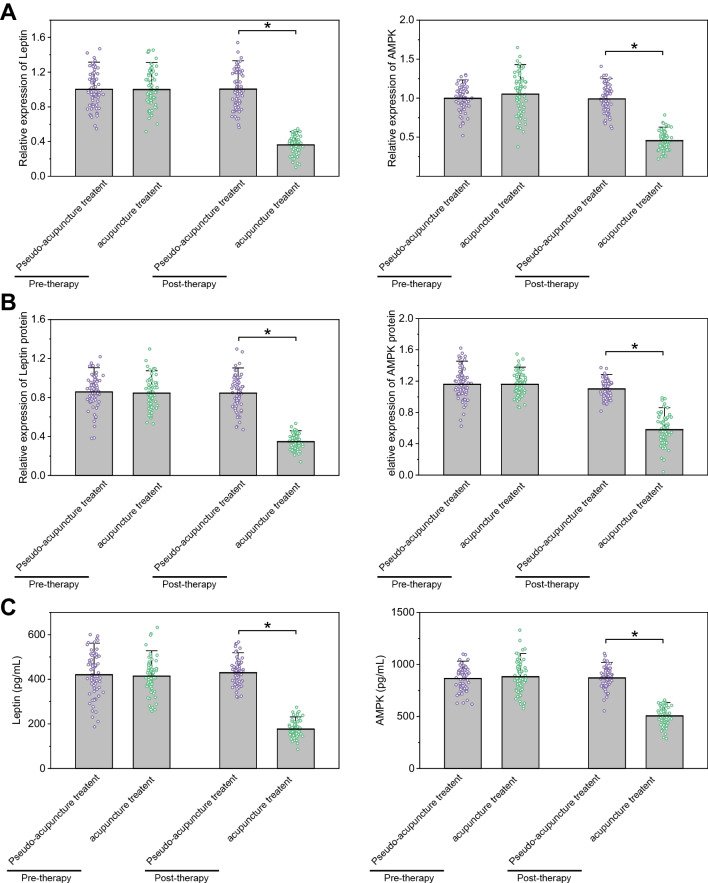
Expression levels of Leptin/AMPK signaling pathway-related factors in peripheral blood of breast cancer patients detected by ELISA after chemotherapy. Note: **A** RT-qPCR was used to detect the expression levels of Leptin and AMPK in the peripheral blood of breast cancer patients before and after acupuncture treatment; **B** Western blot was used to detect the expression levels of Leptin and AMPK in peripheral blood of breast cancer patients before and after acupuncture treatment; **C** ELISA was used to detect the expression levels of Leptin/AMPK signaling pathway-related factors in peripheral blood samples. * indicates *P* < 0.05 compared to pseudo-acupuncture treatment

To investigate the effect of acupuncture treatment on the expression levels of Leptin/AMPK signaling pathway-related factors in breast cancer patients, next, we detected the expression levels of Leptin and AMPK in the peripheral blood of breast cancer patients before and after acupuncture treatment by RT-qPCR, and the results showed (Fig. 3A): before treatment, compared with the sham acupuncture treatment group, the expression levels of Leptin and AMPK expression were not significantly different; after treatment, compared with the sham acupuncture treatment group, the expression of Leptin and AMPK in the acupuncture treatment group was reduced, and consistent results were obtained by Western blot experiments (Fig. 3B), and the expression levels of Leptin/AMPK signaling pathway-related factors in peripheral blood samples were further detected by ELISA, and the results showed that there was no significant difference in the expression of Leptin and AMPK compared with the sham acupuncture treatment group; after treatment, the expression of Leptin and AMPK was reduced compared with the sham acupuncture treatment group (Fig. 3C).

### Acupuncture increases leukocyte counts and reduces mitochondrial DNA mutation rates in breast cancer patients after chemotherapy

**Fig. 4:**
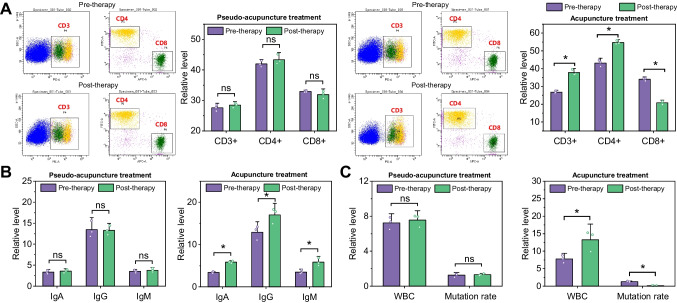
Acupuncture treatment on the expression levels of related factors affecting chemotherapy fatigue in breast cancer patients. Note: **A** column and flow charts of CD3 + , CD4 + , and CD8 + cell expression levels before and after treatment; **B** column charts of IgA, IgG, and IgM expression levels before and after treatment; **C** column charts of white blood cell count and white blood cell mitochondrial DNA mutation rate before and after treatment (where black represents before acupuncture treatment, gray represents after acupuncture treatment, * denotes significant difference, *P* < 0.05)

To investigate the effect of acupuncture treatment on T-cell subsets in the peripheral blood of breast cancer patients before and after chemotherapy, we analyzed T lymphocyte subsets and immunoglobulin levels in peripheral blood before and after treatment using flow cytometry. As shown in Fig. 4A and Table S4, there was no statistically significant difference in T lymphocyte subsets before and after treatment in the sham acupuncture treatment group (all *P* > 0.05); CD3 + and CD4 + levels were significantly higher in the acupuncture treatment group than in the pre-treatment and sham acupuncture treatment groups (all *P* < 0.05), and CD8 + levels were significantly lower than in the pre-treatment and sham acupuncture treatment groups (all *P* < 0.05). No significant changes were observed in the indicators before and after treatment (all *P* > 0.05). The current study found that the activity of CD3 + and CD4 + cells decreased significantly in patients with breast cancer treated with post-operative chemotherapy, while the activity of CD8 + cells increased.

The correlation between cellular immune function and the severity of cancer-caused fatigue has been widely recognized [40, 41]. In this study, CD3 + and CD4 + levels were significantly increased, and CD8 + levels were significantly decreased in the acupuncture treatment group, while no significant changes were observed in the sham acupuncture treatment group, suggesting that acupuncture treatment in post-operative chemotherapy patients with breast cancer is more helpful in regulating T lymphocyte subpopulation levels. To further investigate the effect of acupuncture treatment on the levels of IgA, IgG, and IgM in the peripheral blood of breast cancer patients before and after chemotherapy, we analyzed the levels of IgA, IgG, and IgM in the peripheral blood before and after treatment by immunoturbidimetric method, as shown in Fig. 4B and Table S5, and the results showed that the differences between the levels of IgA, IgG, and IgM in the sham acupuncture treatment group before and after treatment were not statistically IgG is the most basic and the most abundant immunoglobulin in the human body, accounting for 71–75% of the total immunoglobulins, and is the main substance involved in the body’s immune response; IgM is the largest molecular weight and the first immunoglobulin produced by the immune response.

To investigate the effect of acupuncture treatment on peripheral blood leukocytes in breast cancer patients before and after chemotherapy, leukocyte counts were performed using a blood cell analyzer. As shown in Fig. 4C and Table S6, the differences in peripheral blood levels before and after treatment in the sham acupuncture treatment group were not statistically significant (both *P* > 0.05); the peripheral blood leukocyte counts after treatment in the acupuncture treatment group were significantly higher than those before treatment and in the sham acupuncture treatment group (both *P* < 0.05). In addition, the results of the leukocyte mitochondrial DNA mutation rate showed that there was no statistically significant difference in the level of leukocyte mitochondrial DNA mutation rate before and after treatment in the sham acupuncture treatment group (all *P* > 0.05); the leukocyte mitochondrial DNA mutation rate after treatment in the acupuncture treatment group was significantly lower than that before treatment and in the sham acupuncture treatment group (all *P* < 0.05).

## Discussion

Through transcriptome sequencing, we found that the Leptin/AMPK signaling pathway may be a key molecular pathway affecting the occurrence of fatigue in breast cancer patients after chemotherapy. Numerous studies have reported the close relationship between Leptin and AMPK, where Leptin enhances the level of AMPK phosphorylation, activates the AMPK signaling pathway, and promotes the transport of ATP channels [42]. In addition, studies have reported the relationship between Leptin and post-chemotherapy fatigue in breast cancer, where the expression of Leptin increases during fatigue, and the results suggest that Leptin is a potential biomarker for post-chemotherapy fatigue [19]. Furthermore, studies have found that inhibiting the AMPK/PI3K/Akt signaling pathway can alleviate mitochondrial damage and improve tumor-related fatigue [23]. These findings are consistent with the conclusions of our study.

AMPK is an evolutionarily conserved serine/threonine kinase that can induce metabolic changes to maintain the balance of energy production and expenditure within cells. It has been reported that AMPK can directly participate in regulating apoptosis in muscle cells through signaling pathways, including mTOR-ULK1 and FOXO3a [43, 44]. Furthermore, studies have shown that AMPK activation can decrease mitochondrial membrane potential [45]. Additionally, research has demonstrated that Shengmai Fangzheng Injection (SFI), a traditional Chinese medicine formulation, can specifically inhibit the AMPK/PI3K/Akt signaling pathway, suppress muscle cell apoptosis, increase mitochondrial membrane potential, and alleviate mitochondrial dysfunction, thereby improving post-chemotherapy fatigue in cancer patients [23]. Therefore, we believe Leptin may induce phosphorylation and activation of AMPK. When Leptin/AMPK signaling pathway is inhibited by acupuncture treatment, mitochondrial function damage can be reduced, thus improving fatigue after chemotherapy in breast cancer patients. In addition, Leptin, AMPK, and other factors become targets to improve fatigue after chemotherapy in adenocarcinoma patients, thus providing a new theoretical basis for acupuncture therapy to improve fatigue after chemotherapy in adenocarcinoma patients.

Inflammatory markers and fatigue are closely related during the treatment of cancer patients, with high levels of interleukin 6 (IL-6), interleukin 1 (IL-1), and tumor necrosis factor-alpha positively correlating with the degree of fatigue after chemotherapy in cancer patients [46]. These pro-inflammatory factors maintain a relative dynamic balance in the body by regulating secretory signaling pathways in immune and other peripheral cells and facilitating the return of cells to a steady state through the inflammatory process [47]. Stimulated by chemotherapy, tumor cells die, releasing more tumor necrosis factor, inducing more inflammatory responses, and more cytokines are secreted into the peripheral nervous system, signaling to the central nervous system [48, 49]. The central nervous system alters neural processes by sensing inflammation and integrating signals from the peripheral nervous system, resulting in symptoms of fatigue [50–52]. Our results demonstrate that acupuncture can effectively alleviate the fatigue symptoms in breast cancer patients after chemotherapy, and the molecular mechanism may be related to the regulation of the Leptin/AMPK signaling pathway by acupuncture, which is consistent with previous studies.

Furthermore, multiple studies have shown that acupuncture can act on multiple aspects, including the neuroendocrine, immune, metabolic, and hemodynamic systems, to produce a regulatory effect, thereby improving fatigue symptoms in cancer patients [53]. In addition, acupuncture has been proven to significantly improve the immune function of patients with advanced cancer, thereby improving fatigue, depression, and sleep disorders [54]. Studies have also reported that acupuncture treatment can improve fatigue symptoms in breast cancer patients after chemotherapy, and it is a safe and effective treatment for post-chemotherapy fatigue in breast cancer patients [24]. Furthermore, research results have shown that acupuncture treatment can improve post-chemotherapy fatigue in breast cancer patients by regulating the gut microbiota-gut-brain axis [55].

This study also revealed the critical role of the Leptin/AMPK signaling pathway in post-chemotherapy fatigue in breast cancer patients through RNA-Seq sequencing data analysis and protein–protein interaction analysis. The results show that acupuncture is a negative regulatory factor of Leptin, which down-regulates Leptin expression. Conversely, Leptin is a positive regulatory factor of AMPK, and down-regulated Leptin can inhibit the AMPK signaling pathway. Other studies support this finding. Multiple studies have shown that Leptin is related to tumor development and fatigue symptoms and can affect tumor progression and fatigue occurrence through the regulation of metabolism, immune, and neuroendocrine systems, among others [56–58].

Furthermore, in vivo, mouse experiments have shown that acupuncture treatment can reduce Leptin expression levels [59]. In addition, studies have reported the relationship between Leptin and the AMPK signaling pathway, where Leptin can activate the AMPK signaling pathway and promote tumor occurrence and progression [60]. Additionally, we found that the Leptin content in peripheral blood was significantly reduced after treatment [61]. This result is consistent with previous findings that Leptin levels are an important biomarker for tumor progression and fatigue symptoms. Furthermore, previous studies have found that Leptin levels and expression associated with malignant tumors play a vital role in tumor progression and can be used as a diagnostic and prognostic indicator [56, 62].

IgM is the immunoglobulin with the largest molecular weight and the first immune response, which plays a key role in early immune defense but has a lower protective effect than IgG; IgA is the most synthesized globulin, which is widely distributed on the mucosal surface and mainly targets pathogens infecting the mucosa via the respiratory and urinary tracts [63–65]. The levels of IgA, IgG, and IgM were significantly higher in the acupuncture treatment group after treatment than in the pre-treatment and sham acupuncture treatment groups in the results of this study. The amount of change in IgG was even more pronounced, indicating that acupuncture treatment helps the organism’s immune response to function and promotes the synthesis and secretion of immunoglobulins in patients undergoing chemotherapy after breast cancer surgery. Therefore, we hypothesized that acupuncture treatment could help regulate the balance of T lymphocyte subsets and promote immunoglobulin synthesis and secretion in patients treated with post-operative chemotherapy for breast cancer.

Finally, this study found that acupuncture treatment can improve the immune status of T lymphocyte subsets in the peripheral blood of breast cancer patients and reduce the incidence of white blood cell mitochondrial DNA mutations. Our results showed that acupuncture treatment could promote the proliferation ability of peripheral blood leukocytes and that acupuncture is a negative regulatory factor of white blood cell mitochondrial DNA mutation, which can inhibit white blood cell mitochondrial DNA mutation. Various factors influence mitochondrial function, including immunity, metabolism, and oxidative stress. The impairment of these factors can lead to mitochondrial DNA mutations, ultimately affecting mitochondrial function and tumor metabolism [66]. A previous study has shown that acupuncture treatment can improve the immune system function of patients by increasing the antibody level and white blood cell count and protecting the immune organs, among others [67]. In addition, studies have confirmed the significant therapeutic effect and safety of acupuncture treatment for chemotherapy-induced leukopenia [68]. Therefore, we believe that acupuncture treatment can improve the mitochondrial function and tumor metabolism status of cancer patients by improving the immune status and regulating metabolism.

## Conclusion

**Fig. 5:**
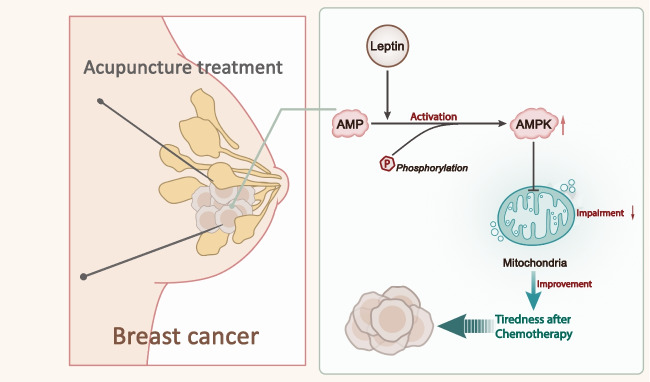
Schematic diagram illustrating the molecular mechanisms underlying the effect of acupuncture treatment on fatigue experienced by breast cancer patients after chemotherapy, through modulating the Leptin/AMPK signaling pathway and regulating mitochondrial function damage

In summary, we can draw the following preliminary conclusions: Leptin is a positive regulator of AMPK, which regulates AMPK phosphorylation, activating the AMPK signaling pathway, and acupuncture treatment can inhibit the Leptin/AMPK signaling pathway. In summary, acupuncture treatment can reduce mitochondrial functional impairment by inhibiting the Leptin/AMPK signaling pathway, thereby improving breast cancer patients. In conclusion, acupuncture treatment can reduce mitochondrial functional impairment by inhibiting the Leptin/AMPK signaling pathway, thereby improving post-chemotherapy fatigue in breast cancer patients (Fig. 5). Our study initially revealed the possible molecular mechanisms underlying the improvement of post-chemotherapy fatigue by acupuncture treatment, which provides a theoretical reference for the clinical management of post-chemotherapy fatigue in cancer patients.

The current study presents an innovative approach to alleviate fatigue in breast cancer patients after chemotherapy by regulating the Leptin/AMPK signaling pathway. The molecular basis of this mechanism is revealed through RNA-Seq transcriptome sequencing and bioinformatics analysis. Moreover, the effectiveness of acupuncture treatment in improving fatigue in breast cancer patients after chemotherapy is validated. Additionally, various methods, including composite fatigue scores, peripheral blood T lymphocyte subsets, and peripheral blood IgA, IgG, and IgM levels, are employed to evaluate fatigue and immune status changes. The results demonstrate a significant improvement in patients’ fatigue symptoms and immune status through acupuncture treatment. However, limitations of the study exist. Firstly, the research employs a small sample size, possibly introducing bias into the results. Secondly, the study lacks in-depth experimental research investigating the mechanism of acupuncture treatment, such as exploring the regulation of the Leptin/AMPK signaling pathway and the underlying molecular mechanisms through cell culture and animal experiments. In addition, specific acupuncture points and stimulation intensity are not adequately investigated. Finally, the study lacks long-term follow-up to determine the long-term effects of acupuncture treatment on breast cancer patients’ fatigue and immune status.

## Supplementary Information

Below is the link to the electronic supplementary material.

Supplementary file1 (DOCX 20 KB)

## Data Availability

The datasets generated and/or analyzed during the current study are available in the manuscript and supplementary materials.

## References

[1] Nasrollahpour H, Khalilzadeh B, Hasanzadeh M, Rahbarghazi R, Estrela P, Naseri A (2023). Nanotechnology-based electrochemical biosensors for monitoring breast cancer biomarkers. Med Res Rev.

[2] El Maouchi P, Fakhreddine O, Shmoury AH, El Zoghbi M, Chamseddine N, AbouZeidane R (2023). Breast cancer knowledge in Lebanese females with positive family history. Medicine (Baltimore).

[3] Sung H, Ferlay J, Siegel RL, Laversanne M, Soerjomataram I, Jemal A (2021). Global Cancer Statistics 2020: GLOBOCAN estimates of incidence and mortality worldwide for 36 cancers in 185 countries. CA Cancer J Clin.

[4] Arnold M, Morgan E, Rumgay H, Mafra A, Singh D, Laversanne M (2022). Current and future burden of breast cancer: global statistics for 2020 and 2040. Breast.

[5] Xia C, Dong X, Li H, Cao M, Sun D, He S (2022). Cancer statistics in China and United States, 2022: profiles, trends, and determinants. Chin Med J (Engl).

[6] Kashyap D, Pal D, Sharma R, Garg VK, Goel N, Koundal D (2022). Global increase in breast cancer incidence: risk factors and preventive measures. Biomed Res Int.

[7] Woolston C (2015). Breast cancer. Nature.

[8] Ponde NF, Zardavas D, Piccart M (2019). Progress in adjuvant systemic therapy for breast cancer. Nat Rev Clin Oncol.

[9] Bower JE (2014). Cancer-related fatigue—mechanisms, risk factors, and treatments. Nat Rev Clin Oncol.

[10] Thong MSY, van Noorden CJF, Steindorf K, Arndt V (2020). Cancer-related fatigue: causes and current treatment options. Curr Treat Options Oncol.

[11] Mohandas H, Jaganathan SK, Mani MP, Ayyar M, Rohini Thevi GV (2017). Cancer-related fatigue treatment: an overview. J Cancer Res Ther.

[12] Agarwal S, Garg R, Minhas V, Bhatnagar S, Mishra S, Kumar V (2020). To assess the prevalence and predictors of cancer-related fatigue and its impact on quality of life in advanced cancer patients receiving palliative care in a Tertiary Care Hospital: a cross-sectional descriptive study. Indian J Palliat Care.

[13] Wayne PM, Lee MS, Novakowski J, Osypiuk K, Ligibel J, Carlson LE (2018). Tai Chi and Qigong for cancer-related symptoms and quality of life: a systematic review and meta-analysis. J Cancer Surviv.

[14] Gernier F, Joly F, Klein D, Mercier M, Velten M, Licaj I (2020). Cancer-related fatigue among long-term survivors of breast, cervical, and colorectal cancer: a French registry-based controlled study. Support Care Cancer.

[15] Bower JE, Bak K, Berger A, Breitbart W, Escalante CP, Ganz PA (2014). Screening, assessment, and management of fatigue in adult survivors of cancer: an American Society of Clinical Oncology clinical practice guideline adaptation. J Clin Oncol.

[16] Yan M, Zhang J, Yang H, Sun Y (2018). The role of Leptin in osteoarthritis. Medicine (Baltimore).

[17] MacDonald IJ, Liu SC, Huang CC, Kuo SJ, Tsai CH, Tang CH (2019) Associations between adipokines in arthritic disease and implications for obesity. Int J Mol Sci 20. 10.3390/ijms20061505 10.3390/ijms20061505 PMC6471239 30917508

[18] Scotece M, Mobasheri A (2015). Leptin in osteoarthritis: focus on articular cartilage and chondrocytes. Life Sci.

[19] Toh YL, Tan CJ, Yeo AHL, Shwe M, Ho HK, Gan YX (2019). Association of plasma leptin, pro-inflammatory adipokines and cancer-related fatigue in early-stage breast cancer patients: a prospective cohort study. J Cell Mol Med.

[20] Gu C, Li T, Jiang S, Yang Z, Lv J, Yi W (2018). AMP-activated protein kinase sparks the fire of cardioprotection against myocardial ischemia and cardiac aging. Ageing Res Rev.

[21] Carling D (2017). AMPK signaling in health and disease. Curr Opin Cell Biol.

[22] Herzig S, Shaw RJ (2018). AMPK: guardian of metabolism and mitochondrial homeostasis. Nat Rev Mol Cell Biol.

[23] Guo W, Liu S, Zheng X, Xiao Z, Chen H, Sun L (2021). Network pharmacology/metabolomics-based validation of AMPK and PI3K/AKT signaling pathway as a central role of Shengqi Fuzheng Injection regulation of mitochondrial dysfunction in cancer-related fatigue. Oxid Med Cell Longev.

[24] Choi TY, Ang L, Jun JH, Alraek T, Birch S, Lu W, et al. (2022) Acupuncture for managing cancer-related fatigue in breast cancer patients: a systematic review and meta-analysis. Cancers (Basel) 14. 10.3390/cancers14184419 10.3390/cancers14184419 PMC9496910 36139579

[25] Wen J, Chen X, Yang Y, Liu J, Li E, Liu J (2021). Acupuncture medical therapy and its underlying mechanisms: a systematic review. Am J Chin Med.

[26] Silva M, Lustosa TC, Arai VJ, Couto Patriota TLG, Lira MPF, Lins-Filho OL (2020). Effects of acupuncture on obstructive sleep apnea severity, blood pressure control and quality of life in patients with hypertension: a randomized controlled trial. J Sleep Res.

[27] Aaronson NK, Ahmedzai S, Bergman B, Bullinger M, Cull A, Duez NJ (1993). The European Organization for Research and Treatment of Cancer QLQ-C30: a quality-of-life instrument for use in international clinical trials in oncology. J Natl Cancer Inst.

[28] Deng G, Chan Y, Sjoberg D, Vickers A, Yeung KS, Kris M (2013). Acupuncture for the treatment of post-chemotherapy chronic fatigue: a randomized, blinded, sham-controlled trial. Support Care Cancer.

[29] Cantarero-Villanueva I, Fernandez-Lao C, Diaz-Rodriguez L, Cuesta-Vargas AI, Fernandez-de-las-Penas C, Piper BF (2014). The Piper Fatigue Scale-Revised: translation and psychometric evaluation in Spanish-speaking breast cancer survivors. Qual Life Res.

[30] Husson O, de Rooij BH, Kieffer J, Oerlemans S, Mols F, Aaronson NK (2020). The EORTC QLQ-C30 summary score as prognostic factor for survival of patients with cancer in the “real-world”: results from the population-based PROFILES registry. Oncologist.

[31] Givan AL (2011). Flow cytometry: an introduction. Methods Mol Biol.

[32] Darzynkiewicz Z, Bedner E, Smolewski P (2001). Flow cytometry in analysis of cell cycle and apoptosis. Semin Hematol.

[33] Ji Z, Wu W, Zhou F, Hu J, Xu Q, Yang W (2021). Effects of sevoflurane exposure on apoptosis and cell cycle of peripheral blood lymphocytes, and immunologic function. BMC Anesthesiol.

[34] Ruan HH, Li GY, Duan N, Jiang HL, Fu YF, Song YF (2018). Frequencies of abnormal humoral and cellular immune component levels in peripheral blood of patients with recurrent aphthous ulceration. J Dent Sci.

[35] Chen HS, Chen J, Cui DL, Zheng YY, Xu AH, Chen G (2007). Effects of a Shuangling Fuzheng anticancer preparation on the proliferation of SGC-7901 cells and immune function in a cyclophosphamide-treated murine model. World J Gastroenterol.

[36] Pierre RV (2002). Peripheral blood film review. The demise of the eye count leukocyte differential. Clin Lab Med.

[37] Yan C, Duanmu X, Zeng L, Liu B, Song Z (2019) Mitochondrial DNA: distribution, mutations, and elimination. Cells 8. 10.3390/cells8040379 10.3390/cells8040379 PMC6523345 31027297

[38] Yang L, Lin X, Tang H, Fan Y, Zeng S, Jia L (2020). Mitochondrial DNA mutation exacerbates female reproductive aging via impairment of the NADH/NAD(+) redox. Aging Cell.

[39] Nagley P, Mackay IR, Baumer A, Maxwell RJ, Vaillant F, Wang ZX (1992). Mitochondrial DNA mutation associated with aging and degenerative disease. Ann N Y Acad Sci.

[40] Li WT, Liu YH, Pan P, Ye SS, Xia Y, Liu AQ (2020). Effects of “Tiaoyi Sanjiao” acupuncture and moxibustion on cancer-induced fatigue and immune function in patients with advanced non-small cell lung cancer. Zhen Ci Yan Jiu.

[41] Schroecksnadel K, Fiegl M, Prassl K, Winkler C, Denz HA, Fuchs D (2007). Diminished quality of life in patients with cancer correlates with tryptophan degradation. J Cancer Res Clin Oncol.

[42] Park SH, Ryu SY, Yu WJ, Han YE, Ji YS, Oh K (2013). Leptin promotes K(ATP) channel trafficking by AMPK signaling in pancreatic beta-cells. Proc Natl Acad Sci USA.

[43] Gui D, Cui Z, Zhang L, Yu C, Yao D, Xu M (2017). Salidroside attenuates hypoxia-induced pulmonary arterial smooth muscle cell proliferation and apoptosis resistance by upregulating autophagy through the AMPK-mTOR-ULK1 pathway. BMC Pulm Med.

[44] Fan J, Yang X, Li J, Shu Z, Dai J, Liu X (2017). Spermidine coupled with exercise rescues skeletal muscle atrophy from D-gal-induced aging rats through enhanced autophagy and reduced apoptosis via AMPK-FOXO3a signal pathway. Oncotarget.

[45] Fan Y, Yang Q, Yang Y, Gao Z, Ma Y, Zhang L (2019). Sirt6 suppresses high glucose-induced mitochondrial dysfunction and apoptosis in podocytes through AMPK activation. Int J Biol Sci.

[46] Ceban F, Ling S, Lui LMW, Lee Y, Gill H, Teopiz KM (2022). Fatigue and cognitive impairment in post-COVID-19 syndrome: a systematic review and meta-analysis. Brain Behav Immun.

[47] Becher B, Spath S, Goverman J (2017). Cytokine networks in neuroinflammation. Nat Rev Immunol.

[48] Simon Q, Grasseau A, Boudigou M, Le Pottier L, Bettachioli E, Cornec D (2021). A pro-inflammatory cytokine network profile in Th1/type 1 effector B cells delineates a common group of patients in four systemic autoimmune diseases. Arthritis Rheumatol.

[49] Rea IM, Gibson DS, McGilligan V, McNerlan SE, Alexander HD, Ross OA (2018). Age and age-related diseases: role of inflammation triggers and cytokines. Front Immunol.

[50] Pedraz-Petrozzi B, Neumann E, Sammer G (2020). Pro-inflammatory markers and fatigue in patients with depression: a case-control study. Sci Rep.

[51] Cadegiani FA, Kater CE (2019). Basal hormones and biochemical markers as predictors of overtraining syndrome in male athletes: the EROS-BASAL study. J Athl Train.

[52] Tomazoni SS, Machado C, De Marchi T, Casalechi HL, Bjordal JM, de Carvalho PTC (2019). Infrared low-level laser therapy (photobiomodulation therapy) before intense progressive running test of high-level soccer players: effects on functional, muscle damage, inflammatory, and oxidative stress markers—a randomized controlled trial. Oxid Med Cell Longev.

[53] Li Y, Yang M, Wu F, Cheng K, Chen H, Shen X (2019). Mechanism of electroacupuncture on inflammatory pain: neural-immune-endocrine interactions. J Tradit Chin Med.

[54] Yang J, Wahner-Roedler DL, Zhou X, Johnson LA, Do A, Pachman DR (2021). Acupuncture for palliative cancer pain management: systematic review. BMJ Support Palliat Care.

[55] Lv Z, Liu R, Su K, Gu Y, Fang L, Fan Y (2022). Acupuncture ameliorates breast cancer-related fatigue by regulating the gut microbiota-gut-brain axis. Front Endocrinol (Lausanne).

[56] Caffa I, Spagnolo V, Vernieri C, Valdemarin F, Becherini P, Wei M (2020). Fasting-mimicking diet and hormone therapy induce breast cancer regression. Nature.

[57] DePeaux K, Delgoffe GM (2021). Metabolic barriers to cancer immunotherapy. Nat Rev Immunol.

[58] Liu L, Shi Z, Ji X, Zhang W, Luan J, Zahr T (2022). Adipokines, adiposity, and atherosclerosis. Cell Mol Life Sci.

[59] Li X, Wu Z, Chen Y, Cai R, Wang Z (2021). Effect of acupuncture on simple obesity and serum levels of prostaglandin E and Leptin in Sprague-Dawley rats. Comput Math Methods Med.

[60] Wu X, Yan Q, Zhang Z, Du G, Wan X (2012). Acrp30 inhibits leptin-induced metastasis by downregulating the JAK/STAT3 pathway via AMPK activation in aggressive SPEC-2 endometrial cancer cells. Oncol Rep.

[61] Zhao S, Kusminski CM, Elmquist JK, Scherer PE (2020). Leptin: less is more. Diabetes.

[62] Kobayashi H, Gieniec KA, Lannagan TRM, Wang T, Asai N, Mizutani Y (2022). The origin and contribution of cancer-associated fibroblasts in colorectal carcinogenesis. Gastroenterology.

[63] Driessen G, van der Burg M (2011). Educational paper: primary antibody deficiencies. Eur J Pediatr.

[64] Li X, Tsuchisaka A, Qian H, Teye K, Ishii N, Sogame R (2015). Linear IgA/IgG bullous dermatosis reacts with multiple laminins and integrins. Eur J Dermatol.

[65] Reddick BK, Crowell K, Fu B (2006) Clinical inquiries: what blood tests help diagnose celiac disease? J Fam Pract 55:1088, 1090, 1093 17137549

[66] Klein HU, Trumpff C, Yang HS, Lee AJ, Picard M, Bennett DA (2021). Characterization of mitochondrial DNA quantity and quality in the human aged and Alzheimer’s disease brain. Mol Neurodegener.

[67] Lertnimitphun P, Zhang W, Fu W, Yang B, Zheng C, Yuan M (2021). Safranal alleviated OVA-induced asthma model and inhibits mast cell activation. Front Immunol.

[68] Jin H, Feng Y, Xiang Y, Zhang Y, Du W, Wasan HS (2020). Efficacy and safety of acupuncture-moxibustion therapy on chemotherapy-induced leukopenia: a systematic review and meta-analysis. Evid Based Complement Alternat Med.

