# Supplementary material for: Acupuncture improves immunity and fatigue after chemotherapy in breast cancer patients by inhibiting the Leptin/AMPK signaling pathway
Source: Support Care Cancer. 2023 Aug 5;31(9):506. doi: 10.1007/s00520-023-07967-1 (PMC10404187; doi:10.1007/s00520-023-07967-1)
Supplement: Supplementary file 1 — Supplementary file1 (DOCX 20 KB) [file 520_2023_7967_MOESM1_ESM.docx]

**Table S1.** RT-qPCR primer sequences

| Gene | Sequences |
| --- | --- |
| Leptin (F) | 5'- TCACACACGCAGTCAGTCTC -3' |
| Leptin(R) | 5'- GAGGTTCTCCAGGTCGTTGG -3' |
| AMPK (F) | 5'- ACAGCCGAGAAGCAGAAACA -3' |
| AMPK(R) | 5'- CTTCACTTTGCCGAAGGTGC -3' |
| GAPDH (F) | 5'-AATGGGCAGCCGTTAGGAAA-3' |
| GAPDH(R) | 5'-GCGCCCAATACGACCAAATC-3' |

**Note:** F: forward; R: reverse.

**Table S2.** Comparison of the total PFSR score and each dimension score between the two groups before and after treatment.

| PFSR scoring | Sham group (N=70) | | Acupuncture group (N=68) | |
| --- | --- | --- | --- | --- |
|  | Pre-therapy | Post-therapy | Pre-therapy | Post-therapy |
| Total points | 5.84±0.86 | 5.20±0.66* | 5.59±0.77 | 3.24±0.70*# |
| Behaviour rating | 5.91±1.33 | 5.19±1.42* | 5.76±1.38 | 3.32±1.69*# |
| Emotional rating | 5.86±1.45 | 5.29±1.28* | 5.51±1.54 | 3.25±1.21*# |
| Feelings rating | 5.74±1.46 | 5.13±1.41* | 5.50±1.34 | 3.15±1.22*# |

Note: * denotes comparison with this group Pre-therapy, *P <* 0.05; # denotes comparison with the sham group post-treatment, *P* < 0.05.

**Table S3.** Comparison of QLQ-C30 total scores and scores of each dimension between the two groups before and after treatment.

| QLQ-C30 scoring | Sham group (N=70) | | Acupuncture group (N=68) | |
| --- | --- | --- | --- | --- |
|  | Pre-therapy | Post-therapy | Pre-therapy | Post-therapy |
| Somatic functions | 36.41±20.61 | 45.74±19.29^*^ | 35.46±12.80 | 63.78±20.47^*#^ |
| Character Functions | 22.13±13.58 | 31.97±15.00^*^ | 20.72±9.87 | 52.78±23.00^*#^ |
| Emotional function | 29.04±11.94 | 38.11±14.01^*^ | 28.69±15.79 | 62.74±25.90^*#^ |
| Cognitive functions | 27.00±9.93 | 37.03±16.10^*^ | 27.10±10.09 | 64.69±12.96^*#^ |
| Social functions | 24.03±8.96 | 35.04±11.00^*^ | 26.03±14.17 | 58.10±26.90^*#^ |
| Tiredness | 78.00±14.38 | 71.57±16.23^*^ | 75.57±15.19 | 43.59±20.68^*#^ |
| Nausea and vomiting | 56.04±14.01 | 45.04±13.03^*^ | 54.40±17.62 | 20.12±9.70^*#^ |
| Pain | 54.00±15.04 | 43.01±14.07^*^ | 49.78±12.11 | 19.04±7.19^*#^ |
| shortness of breath | 48.96±15.96 | 39.00±15.82^*^ | 48.96±13.16 | 20.46±12.50^*#^ |
| Insomnia | 77.39±17.73 | 60.83±19.73^*^ | 72.47±22.41 | 34.22±13.04^*#^ |
| Loss of appetite | 72.49±16.07 | 55.00±14.00^*^ | 77.24±15.99 | 32.28±15.08^*#^ |
| Constipation | 55.93±18.72 | 41.00±12.02^*^ | 53.59±21.61 | 20.60±11.39^*#^ |
| Diarrhoea | 14.06±7.93 | 13.30±7.22 | 16.16±7.01 | 15.29±5.44 |
| Economic hardship | 43.06±18.03 | 41.06±13.97 | 45.72±24.03 | 41.96±10.99 |
| Overall health | 44.99±10.02 | 50.03±17.03^*^ | 39.62±19.30 | 68.40±17.90^*#^ |

Note: * denotes comparison with this group before treatment, *P <* 0.05; # denotes comparison with the sham group after treatment, *P* < 0.05.

**Table S4.** Comparison of T-lymphocyte subpopulation levels between the two groups before and after treatment.

| T-cell subsets | Sham group (N=70) | | Acupuncture group (N=68) | |
| --- | --- | --- | --- | --- |
|  | Pre-therapy | Post-therapy | Pre-therapy | Post-therapy |
| CD3+ | 27.57±1.01 | 28.43±0.76 | 26.77±0.70 | 37.77±1.59^*#^ |
| CD4+ | 45.27±1.16 | 46.87±1.82 | 43.20±1.80 | 54.67±1.08^*#^ |
| CD8+ | 32.93±0.32 | 31.93±1.22 | 34.07±0.84 | 20.87±0.95^*#^ |

Note: * denotes comparison with this group Pre-therapy, *P <* 0.05; # denotes comparison with the sham group post-treatment, *P* < 0.05.

**Table S5.** Comparison of immunoglobulin levels between the two groups before and after treatment.

| Indicators | Sham group (N=70) | | Acupuncture group (N=68) | |
| --- | --- | --- | --- | --- |
|  | Pre-therapy | Post-therapy | Pre-therapy | Post-therapy |
| IgA | 3.36±0.42 | 3.57±0.37 | 3.41±0.19 | 5.84±0.25^*#^ |
| IgG | 13.46±1.92 | 13.29±1.09 | 12.94±1.65 | 17.01±1.82^*#^ |
| IgM | 3.53±0.34 | 3.79±0.41 | 3.48±0.47 | 5.83±0.93^*#^ |

Note: *Compared with Pre-therapy, *P* < 0.05; #Compared with sham group, *P* < 0.05.

**Table S6.** Comparison of peripheral blood white blood cell (WBC) count levels and mitochondrial DNA mutation rate levels between the two groups before and after treatment.

| Indicators | Sham group (N=70) | | Acupuncture group (N=68) | |
| --- | --- | --- | --- | --- |
|  | Pre-therapy | Post-therapy | Pre-therapy | Post-therapy |
| WBC count (%) | 7.24± 0.69 | 7.56 ± 0.71 | 7.78 ± 1.09 | 13.27± 2.97^*#^ |
| Average variation rate (%) | 1.25 | 1.31 | 1.28 | 0.14 ^*#^ |

Note: * denotes comparison with this group before treatment, *P <* 0.05; # denotes comparison with the sham group after treatment, *P* < 0.05.
